# COVID-19 and Tuberculosis—A Global Tale of Hubris and Lessons Unlearned?

**DOI:** 10.3389/fmed.2021.799640

**Published:** 2021-12-22

**Authors:** Francis Drobniewski, Salmaan Keshavjee

**Affiliations:** ^1^Department of Infectious Diseases, Imperial College London, London, United Kingdom; ^2^Department of Global Health and Social Medicine, Harvard Medical School, Boston, MA, United States

**Keywords:** COVID-19, tuberculosis, diagnostics, vaccine distribution, inequality, infection control

The devastating effects of COVID-19 globally have been described in every form of media, with an estimated 252 million cases and over five million deaths (1). Countries around the globe have gone to battle with this organism and faced a heavy toll.

Those who fared better prioritised early diagnostic testing, effective border controls, infection control, and support for the sick and those required to isolate. For the most part, however, while vaccines were being developed we relied on lockdowns and similar quarantine-type approaches as the primary means of stopping transmission of the virus. More alarmingly, we acted as if we had little understanding of airborne transmission of pathogens. Politicians have claimed credit for vaccine success but have dodged responsibility for the early failures and the detrimental effects of isolation on mental health especially for children and adolescents.

Currently, vaccination (in tandem with other strategies) offers the greatest hope for reducing transmission of the virus and high vaccination rates have been achieved in high-income countries such as the USA, UK, countries of the European Union, Israel, etc. Low- and middle-income countries (LMICs), however, have low vaccination rates due to limited vaccine availability. Many LMICs are relying on vaccine supplies through the COVAX facility (COVID-19 vaccines global access) led by the World Health Organisation, GAVI and CEPI (the Coalition for Epidemic Preparedness and Innovations) which has pledged to vaccinate a modest 20% of the population of each LMIC. Specific challenges to vaccination in LMICs, including inadequate cold chain and storage, vaccine hesitancy, and poor transport infrastructure, have all been recognised, but these are irrelevant if there are no vaccines to transport and use.

Although modest efforts to provide free vaccines to LMICs have been made by the USA, UK and others (2), the current strategy is rife with inequity. As of November 15, 2021, 4.1 billion people have received a dose of COVID-19 vaccine (about 53.5% of the world's population). Seventy-five percent of the shots have gone to people in high- and upper-middle-income countries, while <1% has gone to low-income countries (3). As a result, COVID-19 is in danger of becoming an endemic disease of LMICs like tuberculosis (TB). Sadly, as travel restrictions ease, new variants from areas with high transmission will continue to spread, including variants causing serious illness in the non-vaccinated proportion of the population and the wider population if vaccine-escape mutants emerge. Since airborne diseases know no borders, the lack of political will to support vaccination in LMICs is both a scientific blind spot and a moral failure. It also forces us to ask if there are other lessons that we have failed to learn and implement that would reduce the global COVID burden?

Sadly, there is much we should have learned from control efforts for another airborne respiratory disease: tuberculosis. Like COVID-19, TB is mainly transmitted from person to person in the communities where people live and work through droplets and aerosols in the air. For both, more than 40% of people do not exhibit symptoms in the early stages of the disease, which results in more transmission, disease, and death. And while TB does not yet have a highly effective vaccine, it has been curable since the late 1940s. In high-income countries, rates of TB were brought down in the 1960s and 70s using a highly effective strategy: *search* actively and early for new cases in households and communities in order to stop disease transmission; *treat* all forms of the disease rapidly, correctly, and with support for the sick; and *prevent* disease through preventive therapy and infection control (4).

While the search-treat-prevent approach is the bedrock of any airborne epidemic control strategy (5) TB remains the biggest infectious killer of adults, causing the deaths of over 1.5 million people a year and remains the main reason people living with HIV die.

In the divergence between good and bad practises that we have seen for TB and COVID-19, there is much to learn. Sadly, the search-treat-prevent approach did not inform the immediate strategy for addressing COVID-19 transmission for many countries. We lost an opportunity to strengthen the response to COVID-19, forcing reliance on lockdowns as the principle transmission mitigation strategy, and ironically detection and treatment of other diseases, including TB, were compromised. TB notifications dropped by almost 25%, representing the loss of a decade of progress (6).

The first lesson we learned is that it is easy to underestimate the speed with which airborne infections can spread and multiply. What is clear from the history of COVID-19 was the lack of political and health management understanding that cases of an airborne disease might start small in number but can rapidly escalate geometrically to overwhelm health systems. The hubris of those used to seeing small additional increments in patients coming to health facilities as reflective of the amount of disease in the community (e.g., numbers in A&E, number of patients with cancer, etc.) led to poor outcomes, as even short delays in essential measures to limit transmission of the virus cost thousands of lives. Sadly, similar errors were repeated in subsequent waves in the UK, the US, and elsewhere.

The second lesson involves the importance of sufficient diagnostic capacity to identify the infected and sick (see Table 1). TB was overcome in most high-income countries because of the focus on finding cases before widespread transmission could occur. With COVID-19, some countries seemed to have forgotten this important lesson initially. The SARS-CoV-2 genome sequence was available to laboratories at the beginning of 2020. This enabled the early development of diagnostics—including quantitative PCR assays and a variety of high and low throughput assays—and expansion of local and regional laboratory capacity to conduct rapid testing. And while some countries like Germany, South Korea, and Taiwan made wide-spread testing an early priority before the virus became endemic in the community, many others, like the UK and the US, relied initially on clinical symptoms and exposure history as their main metric for disease. Since 40–60% of cases are asymptomatic, it meant that many patients and health care staff were not recognised as having COVID-19. In the UK, patients were discharged from acute hospitals to care homes and community health facilities, seeding the virus amongst those at highest risk, and contributing to an estimated 35,000 excess deaths in the first 2 months of the pandemic in 2020 alone (7). Similarly, care home staff were not prioritised for screening.

**Table 1:** Summary of key areas where TB and COVID 19 strategies may be of mutual relevance.

	**COVID-19 relevant**	**TB relevant**	**COVID-19 missed opportunity**
**Infection control**			
Masks health care workers (HCW)	High	High	Variable
Masks population	Medium	Medium	Importance
Quarantine national (closed airports; supervised isolation)	High	Medium	Missed
Social isolation separation	High	Medium	Variable separation distance chosen
Ventilation and UV-C germicidal lighting	High	High	Missed-importance recognised later
“Lockdown”	Medium-High	Medium	Most effective pre-vaccine approach
Vaccine	High	Medium	Likely most effective approach in absence of effective anti-virals
Financial and food support in isolation	High	High	Essential to maintain isolation
**Major populations affected**			
LMIC	High	High	
People of colour	High	High	
Poor	High	High	
Elderly	High	Medium	
HIV	Medium	High	
DM	High	High	
Renal failure	High	High	
Respiratory failure	Common	Rarer	
Direct anti-pathogen	No	Yes	New antivirals becoming available
Immunomodulation importance	High	Medium	
Mortality (no therapy)	High	High	Good TB therapy available, new antivirals for COVID
Transmission	Contact, respiratory	Respiratory	Stronger evidence supporting Importance of respiratory route for COVID-19 came 6 months into outbreak
Asymptomatic infection	High	High	Covid critical as 40% approx. transmit live virus; TB smaller % and transition to asymptomatic infectious state
**Diagnostics**			
Molecular	High (e.g., QPCR)	High (e.g., PCR)	
Microscopy	Irrelevant	Some value	
Culture	Irrelevant	Essential	
Serology	Valuable	Research needed	Value in COVID dependent on reason for use e.g., rapid, asymptomatic; prior exposure
Whole genomic sequencing/next generation sequencing	High	High	WGS valuable in identifying COVID-19 (e.g., delta variant) or TB clones and likely transmission e.g., nosocomial. Less useful for rapid diagnostics as slow
Point of care	High	High	Near POC as usually clinic/lab based; true high sensitivity/specificity POC needed
**Prevention:**			
Local public health response	Essential	Essential	Many countries e.g., UK did this poorly e.g., contracting private organisations; bypassing skilled public health expertise.

Politicians seemed initially unaware that diagnosis required devices and chemicals, largely made overseas, not just computer modelling. Initially different hospitals and clinical laboratories were competing for the limited global pool of relevant reagents and other materiel particularly for the viral RNA extraction stage for PCR testing. They were also not aware that once the virus moved from small clusters into the community, it became necessary to build systems capable of screening large numbers of people in communities and sharing information about transmission hotspots. This took a long time to happen, and was exacerbated in the UK and elsewhere by a desire to support private sector involvement at the cost of smaller-scale academic and research centres who offered immediate help. The dearth of testing resulted in unintended consequences: for example, where diagnostic capacity for COVID-19 was not expanded, existing molecular diagnostic systems had to be redirected out of immediate necessity, leading to reduced diagnosis of other deadly diseases like TB (8, 9).

Nevertheless, as we emerge from COVID-19, there is an opportunity to expand the use of high throughput platforms for community screening of COVID-19, TB and other deadly respiratory pathogens. Thankfully, the demand for COVID-19 screening encouraged the development of new assays, including point-of-care tests and some low-throughput rapid-turnaround assays. These types of tests helped with infection control in high density institutional settings, especially when coupled with some smart independent thinking leaders who protected the vulnerable early on. A good example was evidenced by outcomes at the Leonard Cheshire care homes in the UK, where early isolation led to only 15 deaths among 2,500 disabled people, far fewer than most other care organisations (10).

The third lesson involves understanding how COVID-19 and TB are both linked to poverty in ways that affect transmission and outcomes (11). The COVID-19 pandemic exacerbated poverty globally, with an additional 88–115 million people pushed into extreme poverty in 2020, and as many as 150 million by 2021 (11). TB has taught us that with infectious diseases it is critical to treat as many people in the communities where they live and work. For this to happen, sick people must be given adequate social and financial support so that they are able to isolate. TB services worldwide have learned how to deliver care to, and monitor, patients in their homes. These lessons were learnt late in the UK and the US, not only compromising the effectiveness of isolation and lockdowns, but putting many vulnerable people at risk.

The fourth lesson is that without investment in prevention, it is difficult to stop airborne epidemics. For TB, we have had preventive/prophylactic strategies that can prevent transmission. Engineering controls that can reduce transmission of viruses and bacteria in public spaces—such as upper room UV-C germicidal air disinfection and improved ventilation (12)—were deployed too late to stop the spread of COVID-19. In many cases, these tools were not deployed at all.

If there is any silver lining in the COVID-19 debacle it is the fifth lesson: that with the right political will, we can develop rapid new diagnostics [and understand their limitations (13)], therapies, and vaccines (e.g., mRNA vaccines). Many of the interventions around COVID-19 allowed health systems to rapidly bring different interventions to scale, overcoming years of health system inertia. Countries and communities have invested in data management systems capable of identifying disease hotspots and sharing information quickly, as well as community clinics and strategies capable of rapidly diagnosing disease and providing care. With so much investment, this infrastructure must not be seen as transient, but as the basis of a more comprehensive form of care delivery for both infectious and chronic diseases.

We have learnt that we must seriously rethink health care delivery globally. COVID-19 exposed inequalities, corruption, and incompetence around the globe and highlighted weaknesses in health system capacity to diagnose and deliver care in the communities where people live and work. This happened almost everywhere, most notably in high-income countries that were thought to be better prepared, like the UK and USA. Some countries (e.g., South Korea, New Zealand, Taiwan, and Germany) drew on their years of experience with TB, and very sensibly built on their existing public health control capacity. They retained key lessons from their TB experience—e.g., the need for rapid diagnosis and identification of at-risk populations, linking people to local care-providers, supporting people who need to be isolated. Others developed alternate systems—like the “Track and Trace” program in the UK—that were expensive, poorly linked to care delivery and did not seem to mitigate unnecessary deaths (14).

We learnt a long time ago that diseases know no borders, and that disease elimination requires a commitment to equity—in diagnostic and therapeutic access, and care delivery. Every infection is a chance for mutation and the creation of variants. While vaccines now offer hope for preventing or at least reducing transmission (as well as mitigating the severity of diseases), this can only happen if they are available to all and are linked to strengthening our community-based systems to search, treat and prevent. Let us not lose the opportunity to put an end to our current pandemics and prevent the next one.

## Author Contributions

All authors listed have made a substantial, direct, and intellectual contribution to the works and approved it for publication.

## Conflict of Interest

The authors declare that the research was conducted in the absence of any commercial or financial relationships that could be construed as a potential conflict of interest.

## Publisher's Note

All claims expressed in this article are solely those of the authors and do not necessarily represent those of their affiliated organizations, or those of the publisher, the editors and the reviewers. Any product that may be evaluated in this article, or claim that may be made by its manufacturer, is not guaranteed or endorsed by the publisher.
